# 

**Published:** 1902-03-01

**Authors:** 


### NOTICE TO CORRESPONDENTS.

All MSS., Letters, Books for Review, and other matters intended for the Editor should be addressed The Editor, "The Hospital," The Hospital Building, 28 & 29 Southampton Street, Strand, W.C.

The Editor cannot undertake to return rejected MS., even when accompanied by stamped directed envelope.

**Notice to Advertisers and Subscribers.**

All Advertisements, Orders for Copies of The Hospital, and Business Communications should be addressed to **THE MANAGER (Not to the Editor),** The Hospital, 28 & 29 Southampton Street, Strand, London, W.C.

The Cost of Subscription, post free, is as follows:—Per week, 2½d.; per quarter, 2s. 8d.; per half-year, 5s. 3d.; per annum, 10s. 6d. Foreign:—Per annum, 15s. 2d., payable, in all cases, in advance.

**NOTICE.—Vol. XXX. of "The Hospital" (April 1901, to September 1901), in handsome cloth binding can now be obtained at the office of this Journal, price, 7s. 6d. Cases for binding the Numbers contained in this Volume 1s. 6d. Index to Vol. XXX., 2½d., post free.**

**The Monthly Part for March is now ready. Price 6d., by post 8d.**

### SCRAPS AND GLEANINGS.

MESSRS. WILSON AND STOCKALL, of Bury, the well-known ambulance builders, are busily employed in executing orders for their patent brougham ambulance for all parts of the country, owing to the small-pox scare.

THE Duke of Portland, as President of the British Lying-in Hospital, is appealing for £7,000 for the purpose of adding to the Hospital a nurses' home with accommodation for 20 nurses and midwives, thus enabling two fresh wards to be opened.

A FUND is being raised, in commemoration of Queen Alexandra's Coronation, to establish pensions and beds in connection with the British Home and Hospital for Incurables, at Streatham, the first charitable institution to receive the support and patronage of Her Majesty.

THE annual report of the Devonshire Hospital (Buxton) states that during 1901 3,039 in-patients and 226 out-patients were admitted; the average daily number of patients in the hospital was 186·1, and the average weekly cost of each patient was 15s. 10¾d.

DR. JANE WALKER, the founder of the Consumption Sanatorium at Nayland, in Suffolk, is appealing for £6,000 to inaugurate a sanatorium in which can be treated patients who cannot pay more than 15s. or £1 a week. If this sum can be raised, the home will, after the initial outlay, be self-supporting.

THE Durham Society for the Cure and Prevention of Consumption is appealing for funds sufficient to provide accommodation for 40 patients. It is estimated that the cost will be about £1,400. The Sanatorium at Stanhope was established two years ago with 12 beds, and the present number of beds is 18.

THE annual report of the Liverpool Hospital for Women showed that during the year 1901, there had been 798 in-patients against 768 in 1900 and 2,468 out-patients against 2,600. The donations included £402 from the Hospital Saturday and Sunday Fund and £228 from the Hospital Century Fund. In spite of these donations the accounts showed a deficit of about £678.

The annual balance-sheet of the Bradford Royal Infirmary showed a deficit of about £900, as compared with £3,918 the previous year, but this difference was accounted for by the fact that in the year under review the amount received from the Joint Hospital Fund was about £2,300 more. The Board of Management are now appealing for funds to, *inter alia*, provide electric lifts (for which a sum of £2,000 will be required), instal a Roentgen ray apparatus and the Finsen light, and properly furnish the operating theatres.

### THE STUDENT OF MEDICINE.

### APPOINTMENTS

**D. Lechmere Anderson**, L.R.C.P., L.R.C.S.Edin., D.P.H., appointed Medical Officer of Health for the Borough of Doncaster and the Parish Councils of Balby-with-Hexthorpe, and Wheatley. **M. F. Cock**, L.R.C.P.Lond., M.R.C.S.Eng., appointed Medical Officer of the Staines Union Workhouse. **Arthur Cooper**, M.R.C.S., appointed Honorary Consulting Surgeon to the Westminster General Dispensary. **Emor R. Cooper**, M.B., Ch.B.Vict. appointed House Surgeon to the Devonshire Hospital, Buxton. **F. S. Dawe**, M.B.Lond., M.R.C.S., appointed Second Assistant Medical Officer of the Kensington Infirmary. **Cradock A. Fry**, M.A., M.B., B.C.Cantab., L.R.C.P., M.R.C.S.Lond., appointed Honorary Assistant Surgeon to the Essex and Colchester Hospital. **G. F. Fulcher**, M.B., C.M.Edin., appointed Medical Officer of Health to the Chingford District Council. **F. Gravely**, M.R.C.S.Eng., appointed Medical Officer of Health for the Chailey Rural District and Medical Officer of the Chailey Workhouse and No. 1 District of the Lewes Union. **R. Humphry**, L.R.C.P.Edin., M.R.C.S.Eng., appointed Medical Officer of Health for the City of Chichester. **T. C. Lawson**, M.R.C.S.Eng., appointed District Medical Officer of the Clutton Union. **H. J. Neilson**, M.D.Glas., appointed District Medical Officer of the Nottingham Union. **W. Wyndham Powell**, F.R.C.S.Eng., appointed Honorary Surgeon to the Westminster Dispensary. **F. E. Reynolds**, M.R.C.S., L.R.C.P., appointed District Medical Officer of the Totnes Union. **Alfred W. Sanders**, M.D.Lond., F.R.C.S., appointed District Surgeon for Pretoria, Transvaal, and Surgeon for the Pretoria Hospital. **Charles Lane Sansom**, F.R.C.S.Edin., L.S.A., appointed District Health Officer of the Transvaal. **Robert Fox Symons**, M.R.C.S., L.R.C.P., appointed Medical Officer of Health for Pretoria and District and Assistant Medical Officer of Health for the Transvaal. **J. W. Wyncoll**, M.B., C.M.Edin., appointed Resident Medical Officer to the Westminster Dispensary.

